# Supplementary material for: Copper nanoparticles encapsulated in zeolitic imidazolate framework-8 as a stable and selective CO2 hydrogenation catalyst
Source: Nat Commun. 2024 Mar 6;15:2045. doi: 10.1038/s41467-024-46388-4 (PMC10918174; doi:10.1038/s41467-024-46388-4)
Supplement: Supplementary file 1 — Supplementary Information [file 41467_2024_46388_MOESM1_ESM.pdf]

Supporting information of

# Copper nanoparticles encapsulated in zeolitic imidazolate framework-8 as a stable and selective $CO_2$ hydrogenation catalyst

Vijay K. Velisoju[a], Jose L. Cerrillo[b], Rafia Ahmad,[b] Hend Omar Mohamed[a], Yerrayya Attada[a], Qingpeng Cheng[b,c], Xueli Yao[a], Lirong Zheng[d], Osama Shekhah[c], Selvedin Telalovic[b], Javier Narciso[e], Luigi Cavallo[b], Yu Han[b,c], Mohamed Eddaoudi[c], Enrique V. Ramos-Fernández[b,e], Pedro Castaño[a,f,*]

[a] Multiscale Reaction Engineering, KAUST Catalysis Center (KCC), King Abdullah University of Science and Technology (KAUST), Thuwal, 23955-6900, Saudi Arabia.

[b] KAUST Catalysis Center (KCC), King Abdullah University of Science and Technology (KAUST), Thuwal, 23955-6900, Saudi Arabia.

[c] King Abdullah University of Science and Technology (KAUST), Physical Sciences and Engineering Division, Advanced Membranes and Porous Materials (AMPM) Center, Thuwal 23955-6900, Saudi Arabia

[d] Beijing Synchrotron Radiation Facility, Institute of High Energy Physics, Chinese Academy of Sciences, Beijing, 100049 China

[e] Laboratorio de Materiales Avanzados, Departamento de Química Inorgánica – Instituto Universitario de Materiales de Alicante, Universidad de Alicante, Apartado 99, E-03080 Alicante, Spain.

[f] Chemical Engineering Program, Physical Science and Engineering (PSE) Division, KAUST, Thuwal, Saudi Arabia.

*Corresponding author: pedro.castano@kaust.edu.sa

**Results**

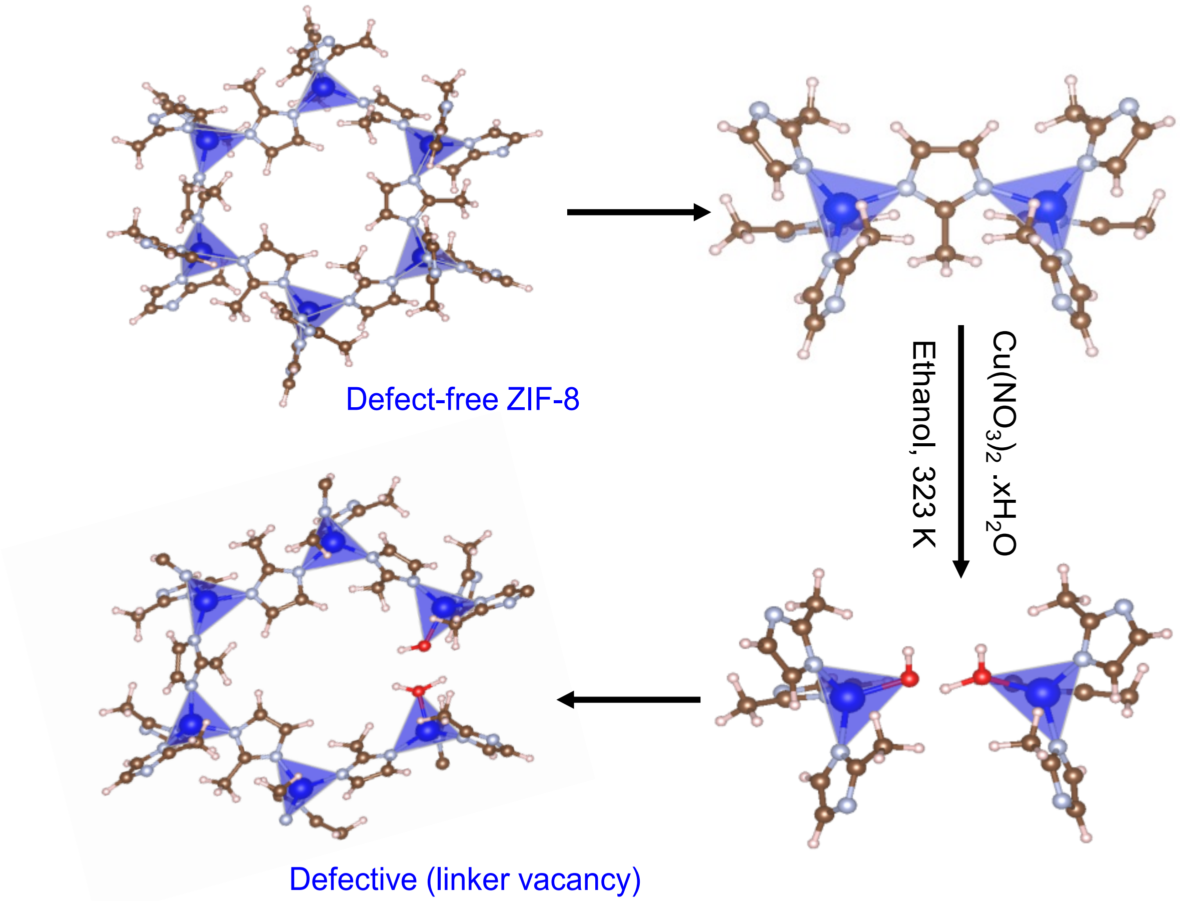


**Fig. S1.** Missing linker (2-methylimidazole) replaced by –OH/$OH_2$ in the Zn nodes of the MOF that are active for ion exchange (Cu capture). The colors used to represent atoms are as follows: N (cyan), Zn (blue (tetrahedrons)), C (dark brown), O (red), and H (light pink).

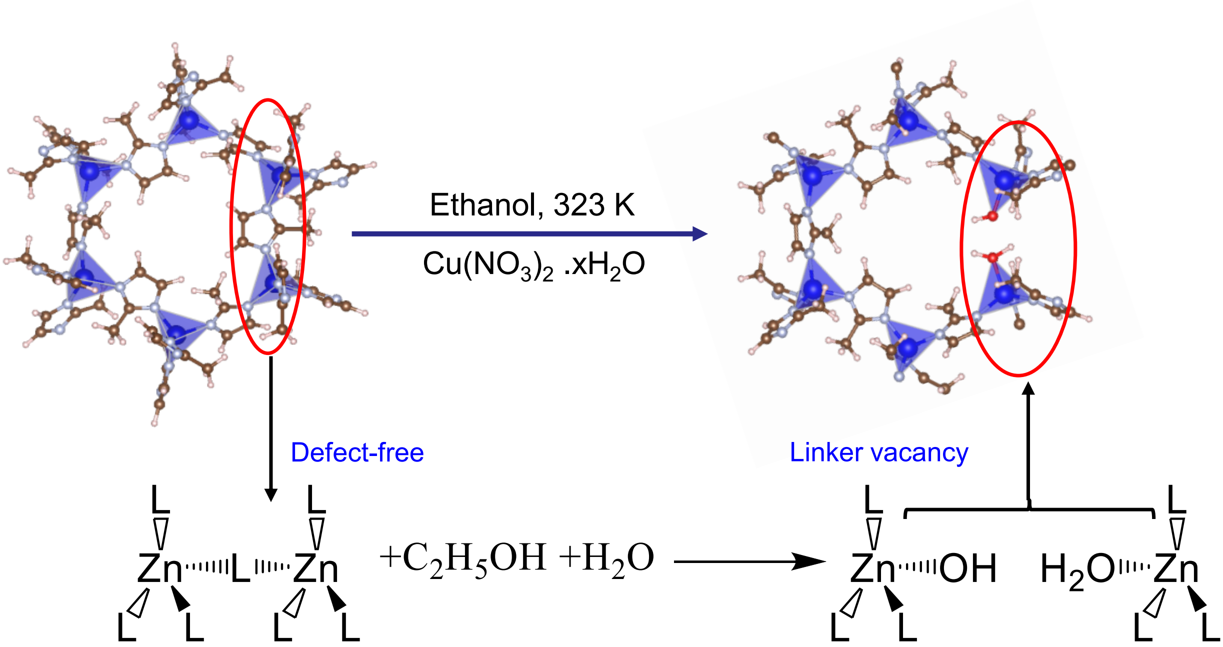


**Fig. S2.** Using defective nodes to deposit Cu species. A linker missing (2-methylimidazole) from the ZIF-8 structure is replaced by–OH/$OH_2$ species, which are active for Cu ion exchange into the MOF.[1,2] The colors used to represent atoms are as follows: N (cyan), Zn (blue (tetrahedrons)), C (dark brown), O (red), and H (light pink).

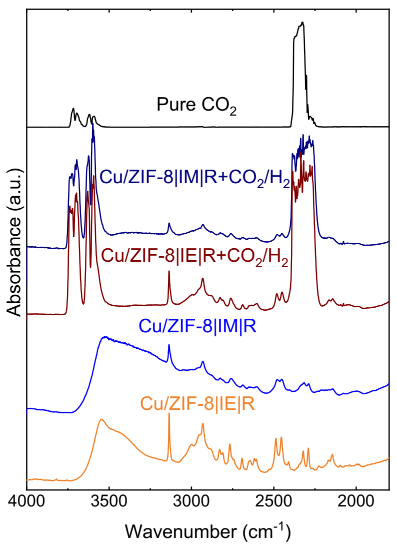


**Fig. S3.** In situ DRIFT spectra of Cu/ZIF-8|IE|, and Cu/ZIF-8|IM| catalysts before and after adsorption of $CO_2$+$H_2$ (1:3) under 25 bar after reduction at 523 K for 1 h.

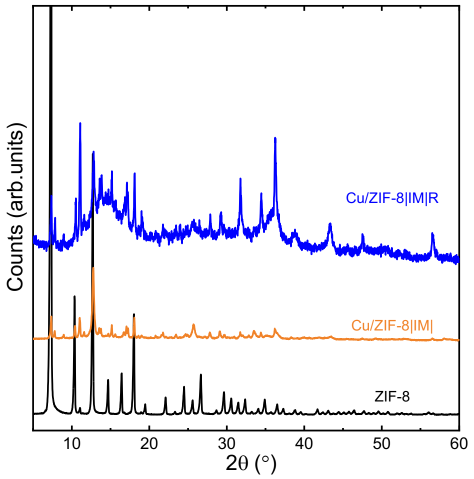


**Fig. S4.** Powder XRD patterns of ZIF-8 and Cu/ZIF-8|IM| catalysts before and after reduction at 523 K.

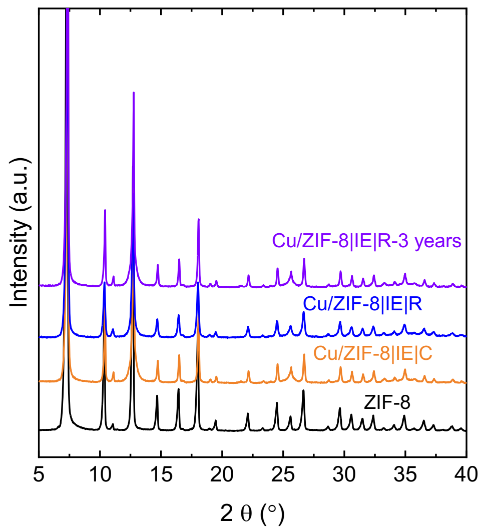


**Fig. S5.** Powder XRD patterns of ZIF-8 and Cu/ZIF-8|IE| (after ion-exchange), Cu/ZIF-8|IE|R (after reduction at 523 K), and Cu/ZIF-8|IE|R after 3 years storage in a glass vial.

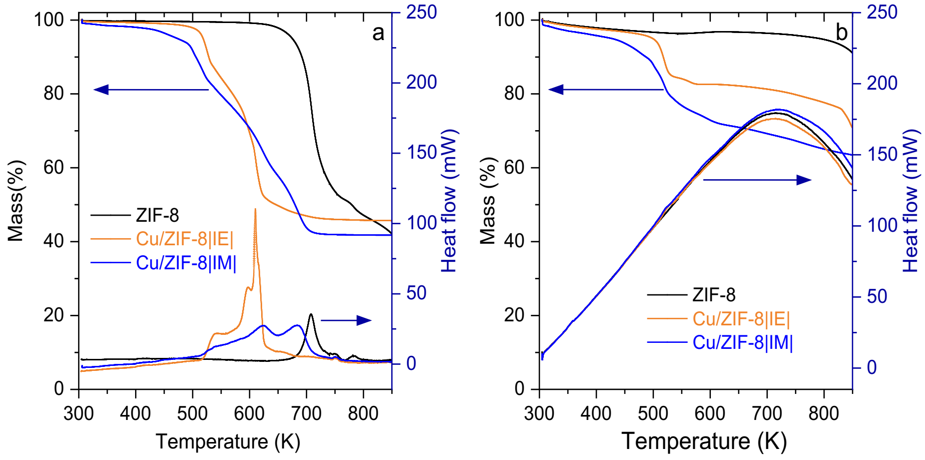


**Fig. S6.** Thermogravimetric analysis (TGA) and differential thermal analysis (DTA) profiles of ZIF-8 and Cu/ZIF-8|IE|, and Cu/ZIF-8|IM| materials after preparation carried out in different atmospheres (a) zero air, and (b) 6%$H_2$/Ar.

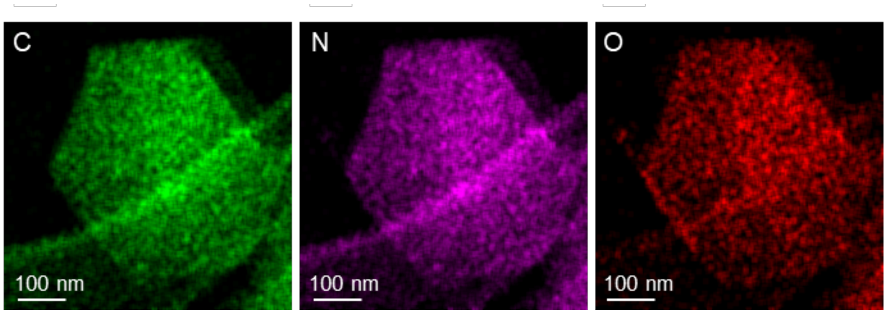


**Fig. S7.** HAADF – STEM – EDX analysis of ZIF-8 sample at different resolutions. The colors used to represent atoms are as follows: C (green), N (purple), and O (red).

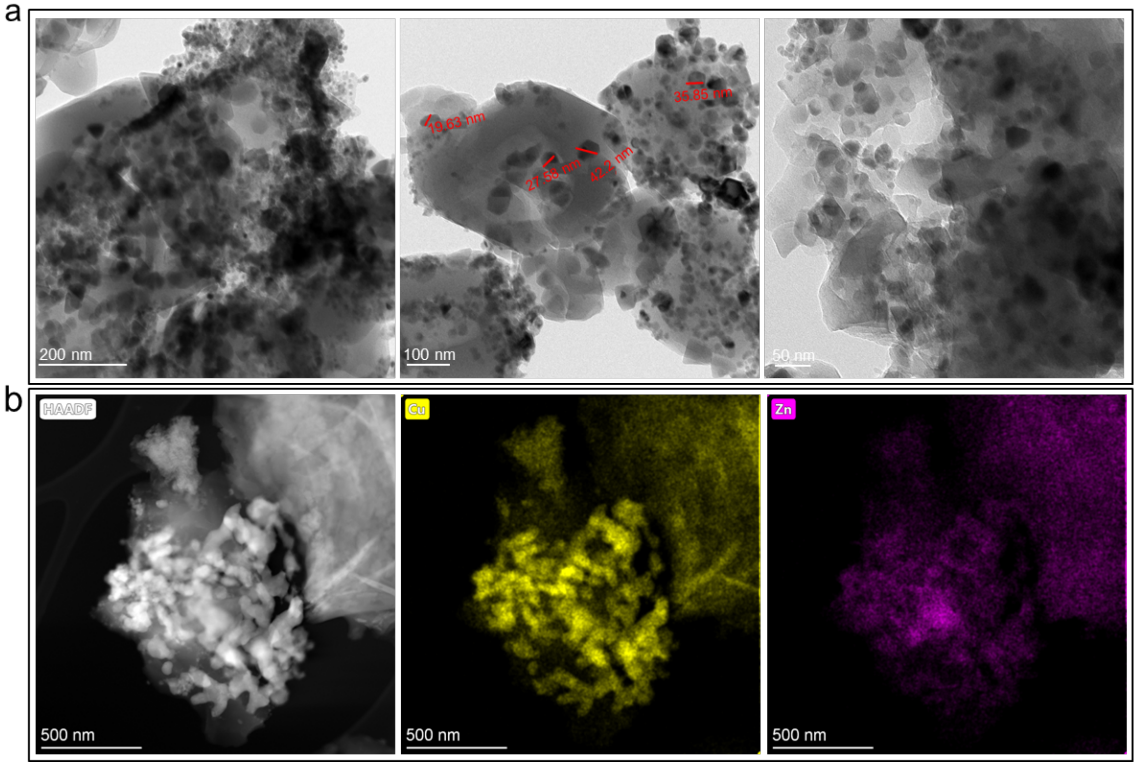


**Fig. S8.** (a) TEM and (b) HAADF-STEM-EDX images of Cu/ZIF-8|IM|R sample after reduction at 523 K at different resolutions. The colors used to represent atoms are as follows: Cu (yellow), Zn (purple).

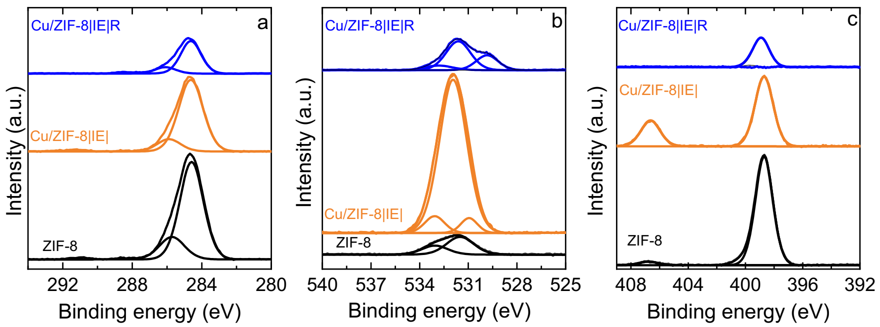


**Fig. S9.** XPS spectra of ZIF-8 and Cu/ZIF-8|IE| samples before and after reduction at 523 K a) C 1s, b) O 1s, and c) N 1s.

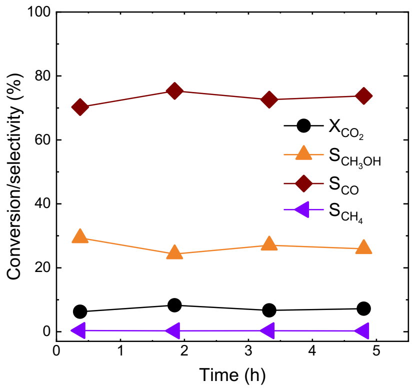


**Fig. S10**. $CO_2$ hydrogenation to methanol performance over the Cu/ZIF-8|IE|R-100 h catalyst under 25 bar. Reaction conditions: 80%$H_2$/20%$CO_2$ feed; T = 523 K, P = 25 bar, GHSV = 15,750 $h^{-1}$.

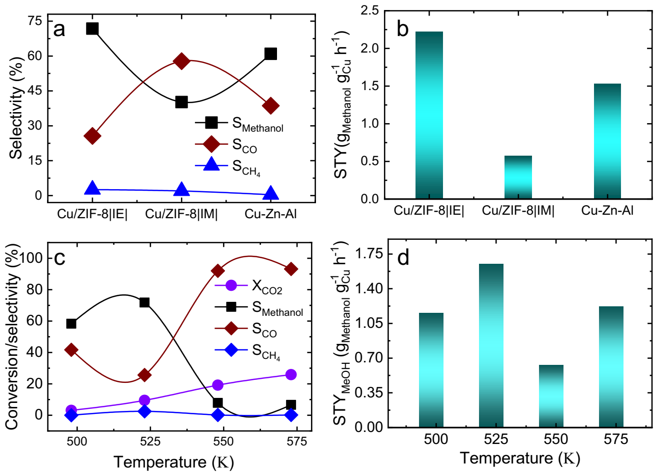


**Fig. S11.** (a) Comparison of $CO_2$ to methanol performance of the Cu-loaded ZIF-8 catalysts in this study with that of the commercial Cu-Zn-Al catalyst, and (b) corresponding space-time-yields. Reaction conditions: 80%$H_2$/20%$CO_2$ feed; T = 523 K, P = 50 bar, Reaction time: 6 h, GHSV = 15,750 $h^{-1}$. (c) Influence of reaction temperature on product selectivity and (d) their corresponding methanol space-time yields over the Cu/ZIF-8|IE| catalyst. Reaction conditions: 80% $H_2$/20% $CO_2$ feed, P = 50 bar, GHSV = 15,750 $h^{-1}$.

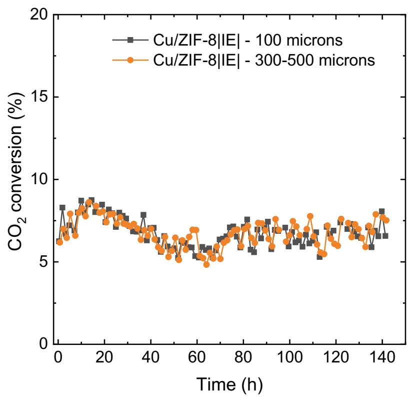


**Fig. S12.** $CO_2$ hydrogenation to methanol performance over the size-specific Cu/ZIF-8|IE|-100 h catalysts over time. Reaction conditions: 80%$H_2$/20%$CO_2$ feed; T = 523 K, P = 50 bar, GHSV = 15,750 $h^{-1}$.

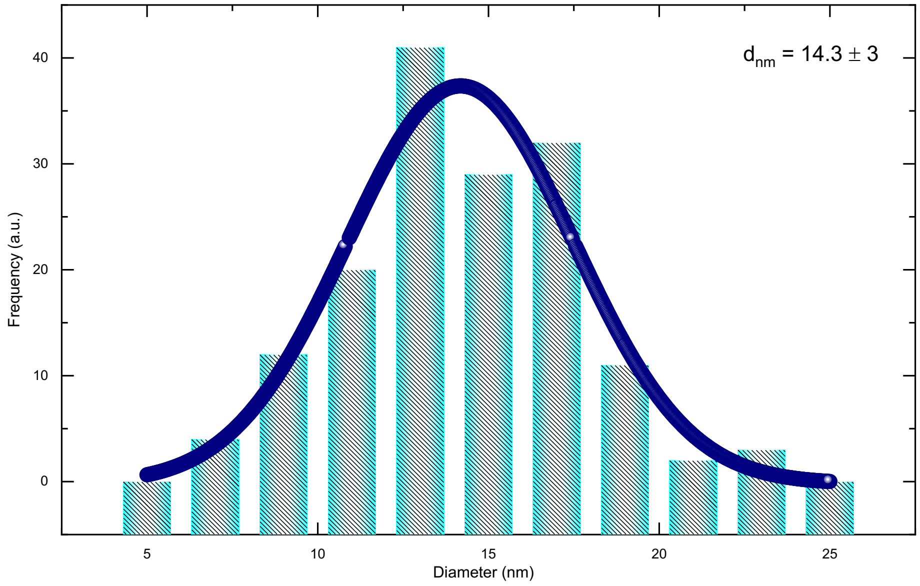


**Fig. S13.** Particle size distribution obtained from TEM analysis of Cu/ZIF-8|IE|R-100 h catalyst.

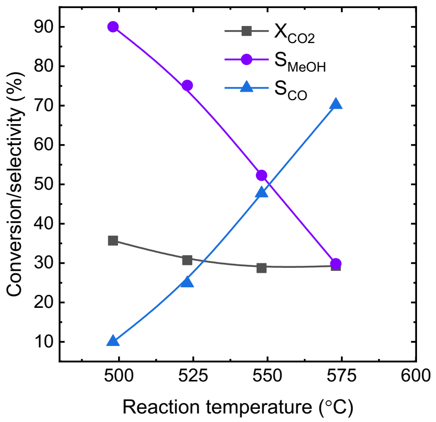


**Fig. S14.** Effect of reaction temperature on the thermodynamic equilibrium of $CO_2$ conversion and product selectivity for CO and methanol products at 50 bar with 80%$H_2$/20%$CO_2$.

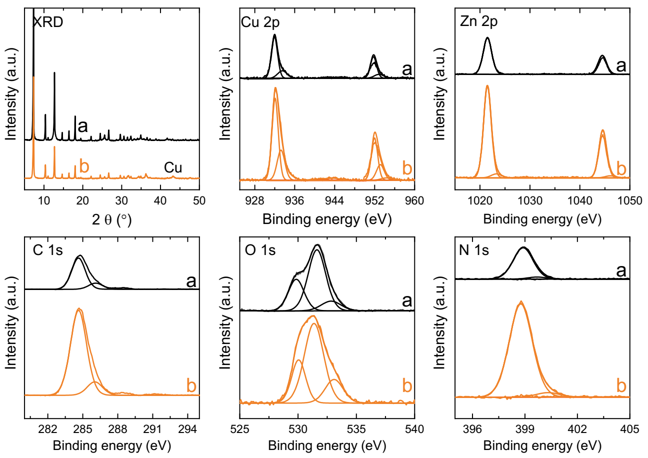


**Fig. S15.** Powder XRD patterns, XPS spectra of (a) Cu/ZIF-8IIEIR and (b) Cu/ZIF-8IIEIR-100 h catalyst.

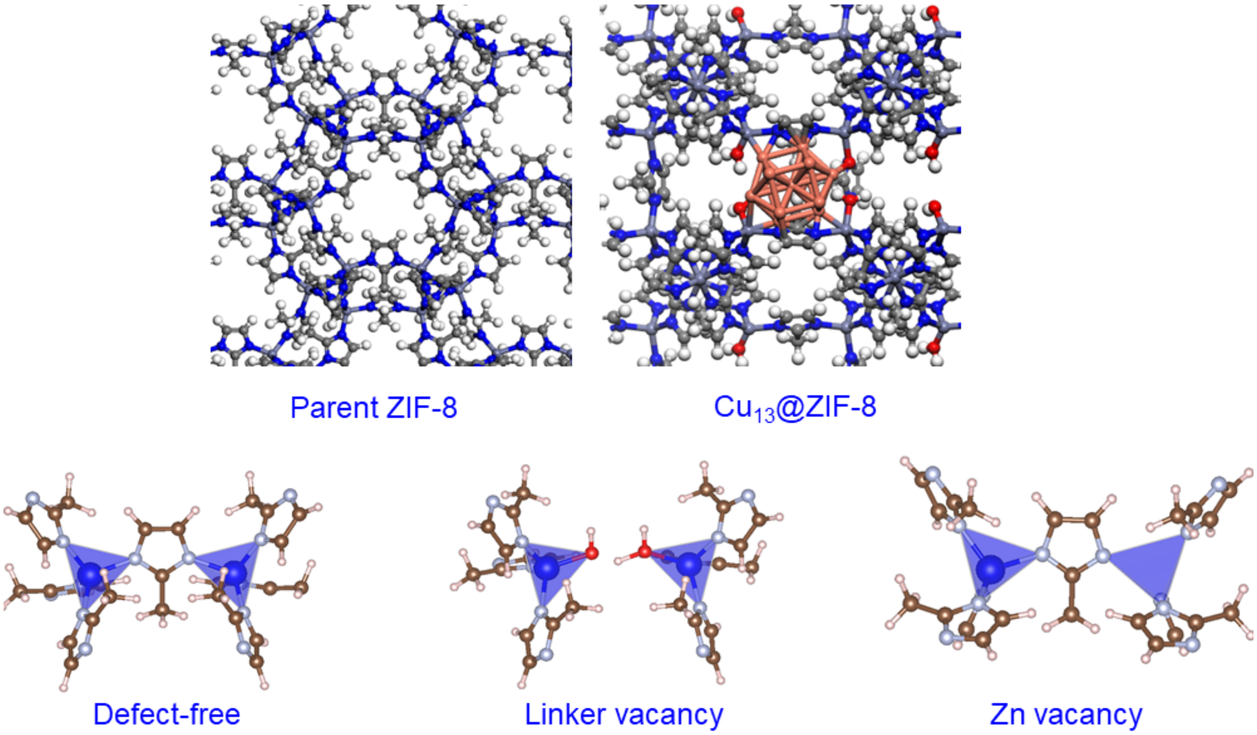


**Fig. S16.** (top) Density functional theory (DFT) optimized structures for $CO_2$ adsorption on ZIF–8 without any linker vacancies (left image) and a $Cu_{13}$ cluster-loaded ZIF-8 with linker vacancies (right image). (bottom) Three different possible connections of the ZIF-8 network in defect-free ZIF-8 (left), fully Cu-exchanged (middle), and partially Cu-impregnated ZIF-8 systems (right).

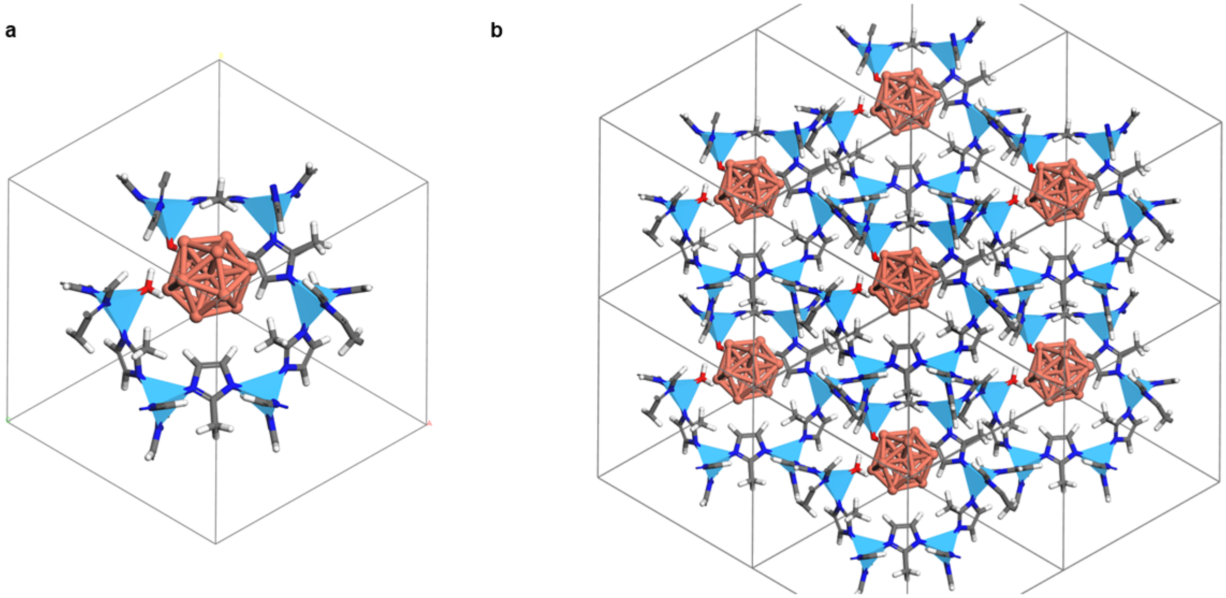


**Fig. S17.** A 13-atom icosahedral Cu nanoparticle grafted at the 2-methylimidazole linker vacancy (–OH/–$OH_2$ node) in (a) The primitive cell and (b) repeated supercell (to view the periodic structure) of sodalite ZIF-8. Brown, blue, gray, light blue, red and white colors are used to denote Cu, N, C, Zn, O and H atoms, respectively.

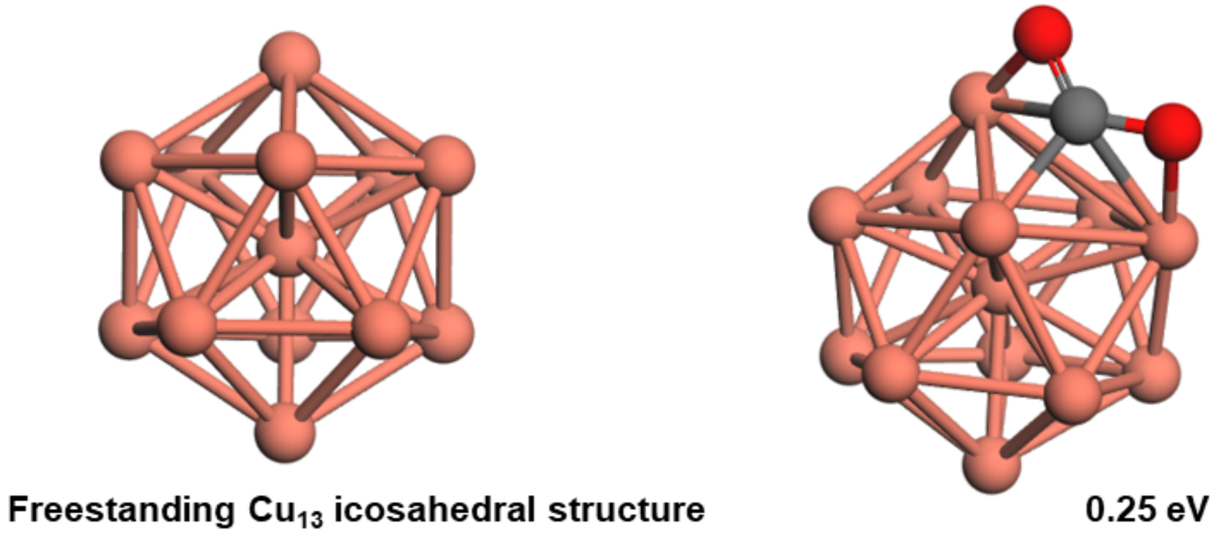


**Fig. S18.** A13 atom Cu icosahedral structure and $CO_2$ adsorption geometry on the free-standing cluster and free energy of $CO_2$ adsorption at 523 K. Brown, red and black colors are used to denote Cu, O and C atoms, respectively.

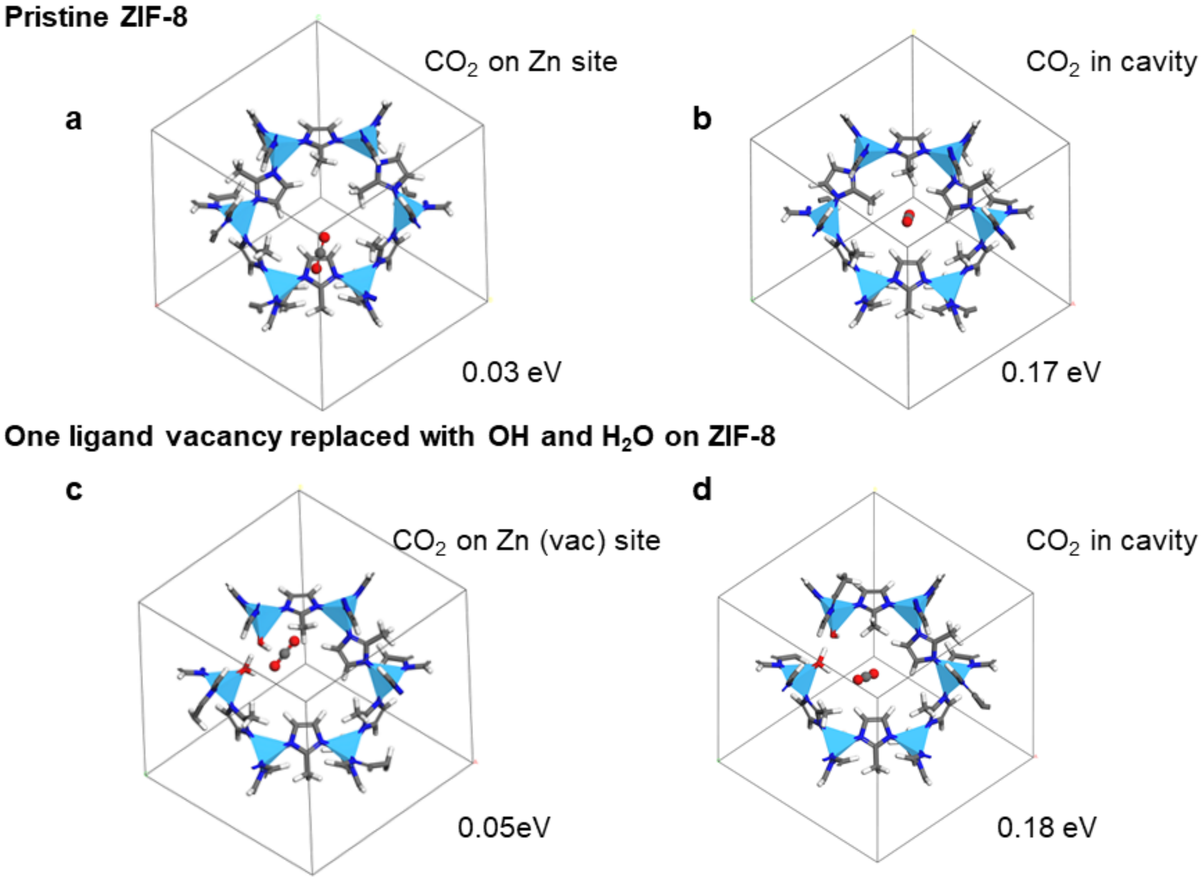


**Fig. S19.** $CO_2$ adsorption geometries and free energies on pristine primitive cell of sodalite ZIF8 for a) near a Zn site and (b) inside the hollow cavity, and on ZIF8 with one 2-methylimidazole linker vacancy substituted by $H_2O$ and OH (c) near the Zn site of vacancy and (d) inside the hollow cavity. Blue, gray, light blue, red and white colors are used to denote N, C, Zn, O and H atoms, respectively. Fee energies of $CO_2$ adsorption are calculated at 523 K.

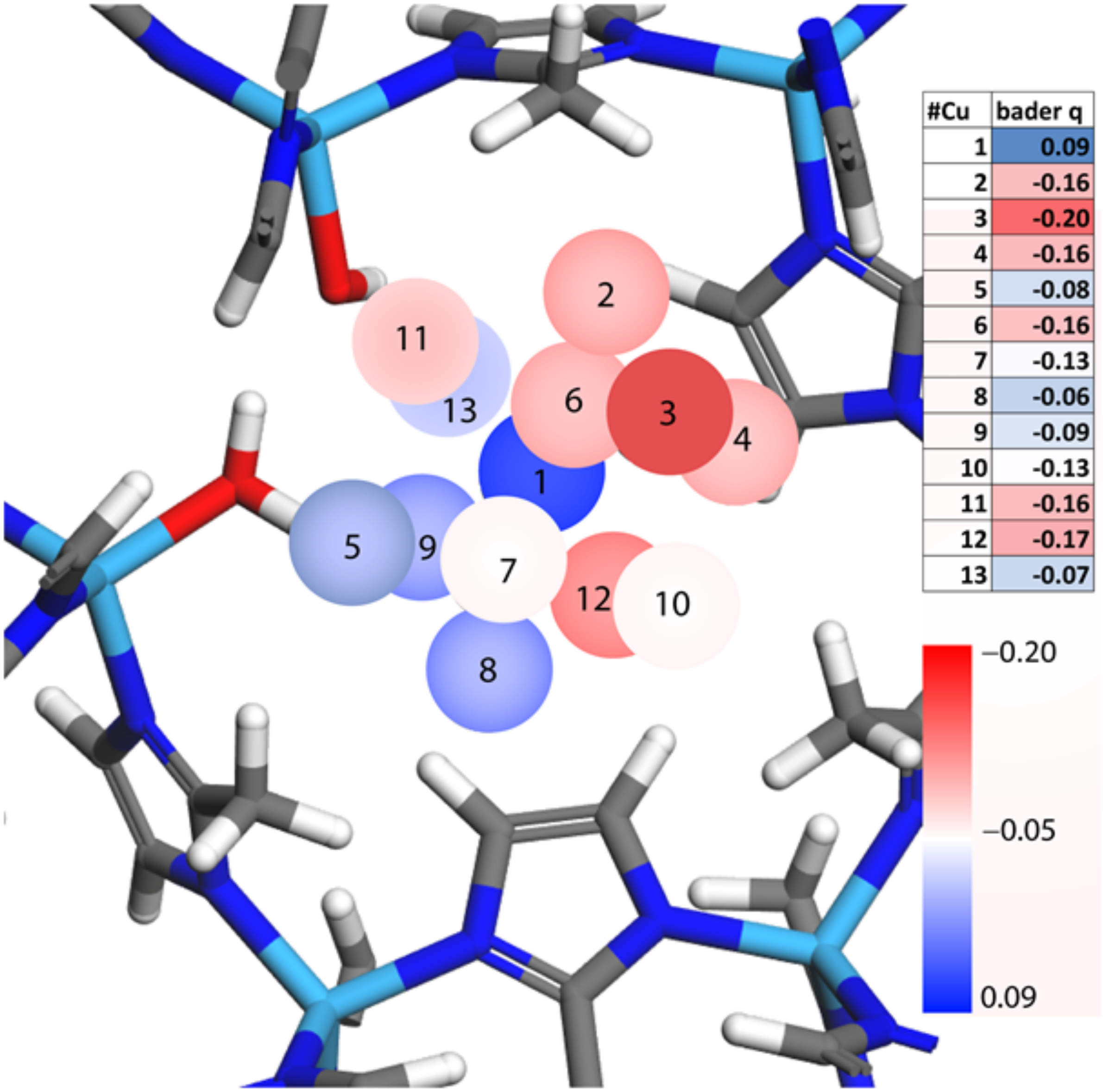


**Fig. S20.** Bader charge analysis for the 13-atom Cu cluster in the ZIF-8 cavity at the linker vacancy site. Blue, gray, light blue, red and white colors are used to denote Cu, N, C, Zn, O and H atoms, respectively. The Cu atoms are numbered are expressed in a heatmap to show the changes in Bader charge for each of them.

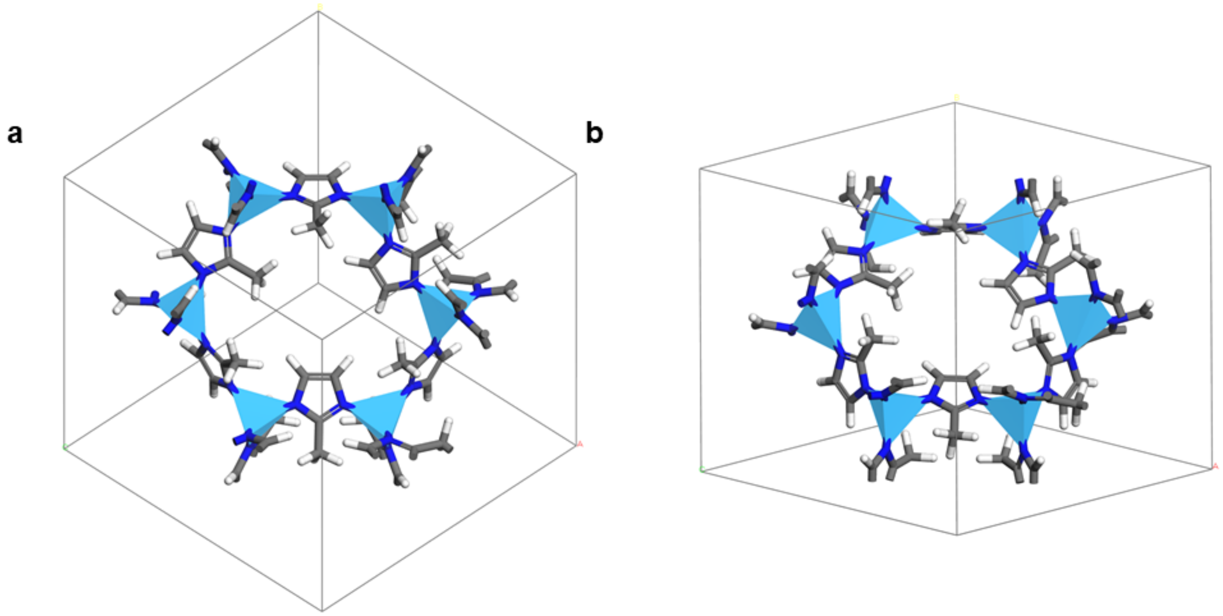


**Fig. S21.** Primitive cell of sodalite ZIF8 with 2-methylimidazole linkers in two different viewing angles (a and b). Blue, gray, light blue and white colors denote N, C, Zn and H atoms, respectively.

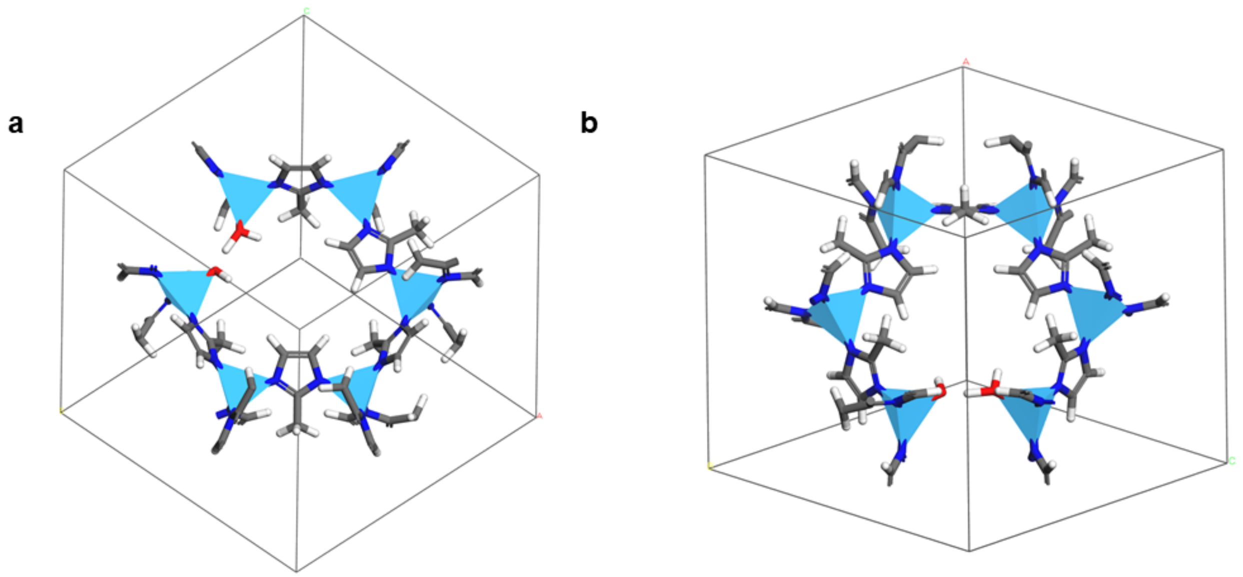


**Fig. S22.** Primitive cell of sodalite ZIF8 with one 2-methylimidazole linker vacancy substituted by $H_2O$ and OH creating the –OH/–$OH_2$ node in two different viewing angles (a and b). Blue, gray, light blue, red and white colors denote N, C, Zn, O and H atoms, respectively.

Table S1. Physico-chemical characteristics of the catalysts.

| Catalyst | Element (wt%)[a] | | | $S_{BET}$ ($m^2/g$)[b] | Crystallite size (nm)[c] | Uptake ($\mu mol/g_{cat}$) | | O/Zn ratio[f] |
|---|---|---|---|---|---|---|---|---|
| | Cu | Zn | Al | | | $CO_2$[d] | $NH_3$[e] | |
| ZIF-8 | - | 27.4 | - | 1167 | 62 | 41 | 77 | 0.26 |
| Cu/ZIF-8\|IE\|R | 12.1 | 19.3 | - | 880 | 81 | 2541 | 167 | 0.79 |
| Cu/ZIF-8\|IM\|R | 12.9 | 14.7 | - | 25 | 67 | 649 | 1101 | 0.84 |
| Cu-Zn-Al | 43.7 | 15.6 | 3.6 | 79 | - | - | - | - |

[a] Determined from ICP-OES analysis
[b] Obtained from $N_2$-sorption analysis
[c] Calculated from powder XRD patterns w.r.t. ZIF-8 (110) plane
[d] Calculated from $CO_2$-TPD analysis
[e] Calculated from $NH_3$-TPD analysis
[f] Obtained from XPS analysis

Table S2. Relative surface compositions obtained from XPS analysis.

| Catalyst | Cu/Zn | O/Cu | Cu/O | O/(O+Zn+Cu+N)*100 |
|---|---|---|---|---|
| ZIF-8 | - | - | - | 13.7 |
| Cu/ZIF-8\|IE\| | 0.97 | 0.94 | 1.06 | 28.9 |
| Cu/ZIF-8\|IE\|R | 0.46 | 1.69 | 0.59 | 31.2 |
| Cu/ZIF-8\|IM\| | 0.45 | 1.88 | 0.53 | 30.6 |
| Cu/ZIF-8\|IE\|R-100 h | 0.46 | 1.02 | 0.98 | 20.3 |

Table S3. Particle size and crystallite size of copper from different techniques.

| Catalyst | Particle size (nm)[a] | Crystallite size (nm)[b] | Active particle size (nm)[c] |
|---|---|---|---|
| Cu/ZIF-8\|IE\|R-100 h | 14.2 ± 3 nm | 15.8 nm | 16.6 nm |

[a] Average particle size estimated from TEM analysis.

[b] Crystallite size calculated from Scherrer equation

[c] Active particle size obtained from $N_2O$ pulse titration method.

Table S4. Comparison of the present study with previously reported systems for methanol production.

| Catalyst | $S_{MeOH}$ (%) | Productivity ($g_{MeOH}$ $g_{metal}^{-1}$ $h^{-1}$) | TOF ($s^{-1}$) | Time on stream | Ref. |
|---|---|---|---|---|---|
| Cu/UiO-bpy | 100 | 0.0025 (Cu) | 0.0026[a] | 100 h | [3] |
| Zn/MOF-808 | 99 | 0.19 (Zn) | - | 100 h | [4] |
| Cu/UiO-66 | 50 | 2.35 (Cu) | 0.0015[a] | 50 h | [5] |
| Cu⊂UiO-66 | 100 | - | 0.0037[a] | 8 h | [6] |
| Cu-Zn-Al | 61 | 1.47 (Cu) | 0.0057 | >100 h | Commercial |
| Cu/ZIF-8\|IE\|R | 90 | 2.27 (Cu) | 0.0173 | >150 h | This work |

[a] As given in the published data.
